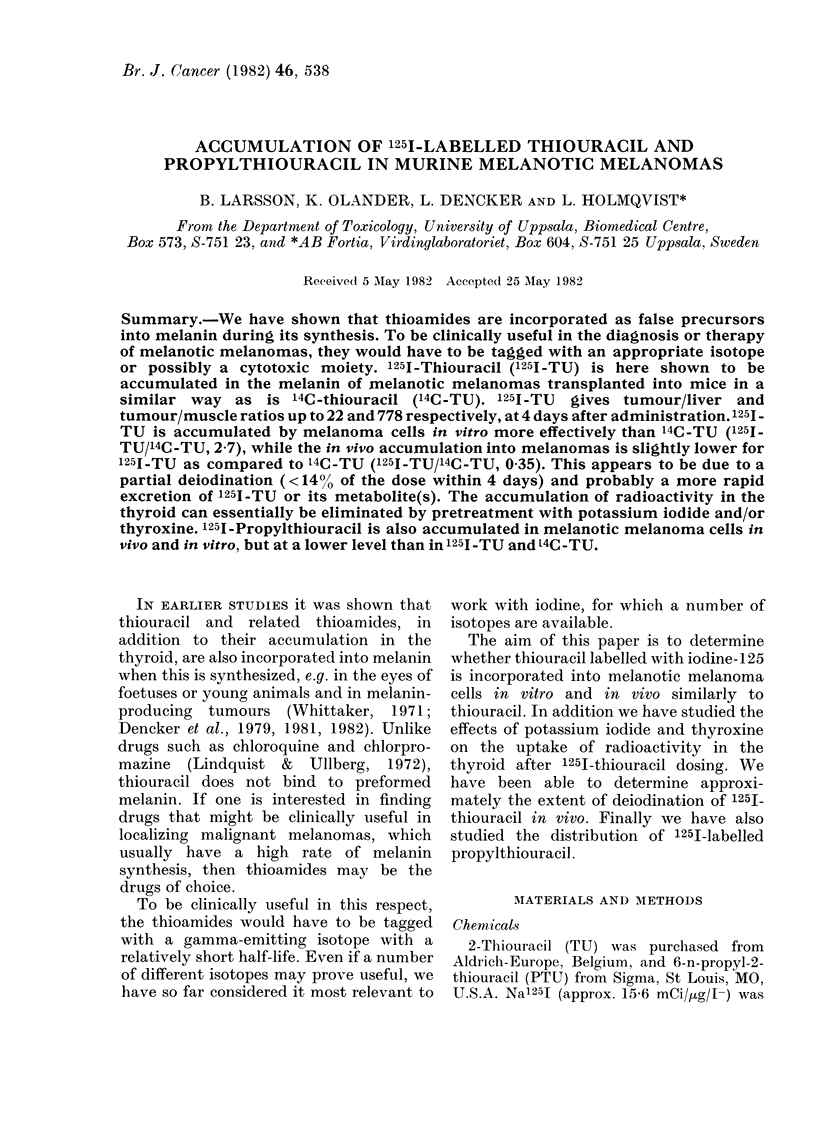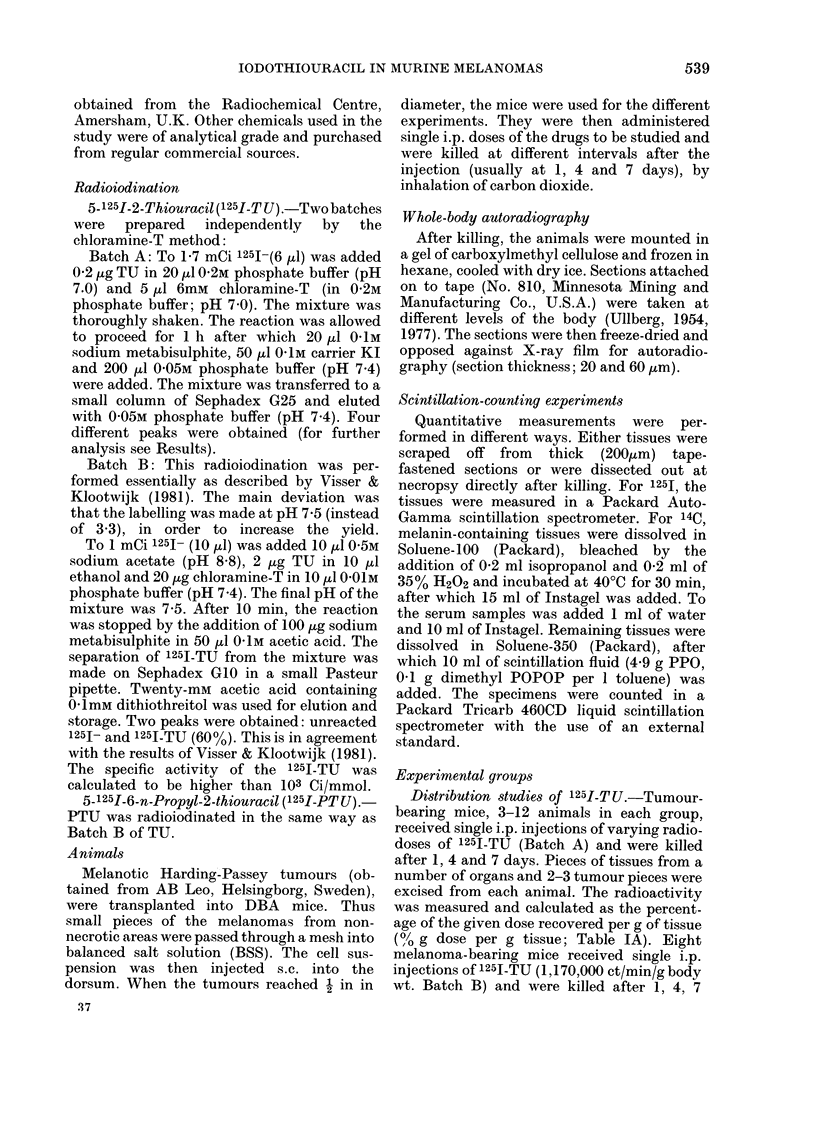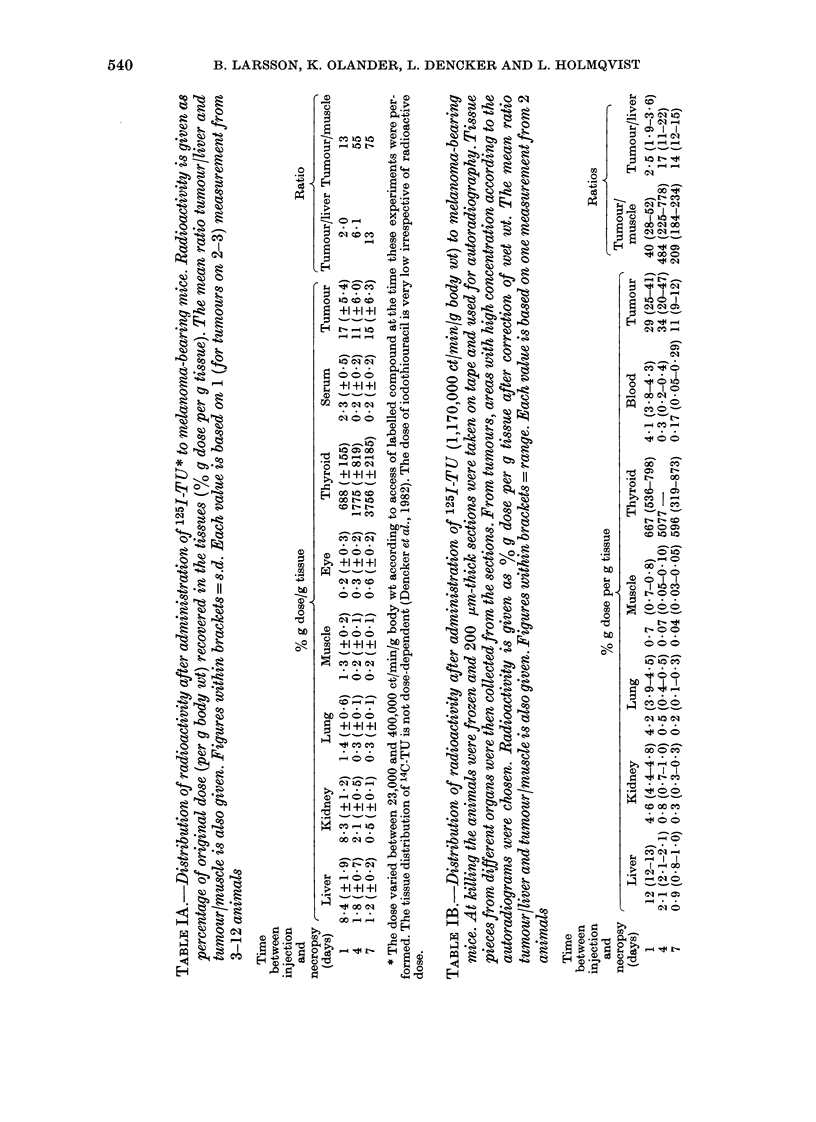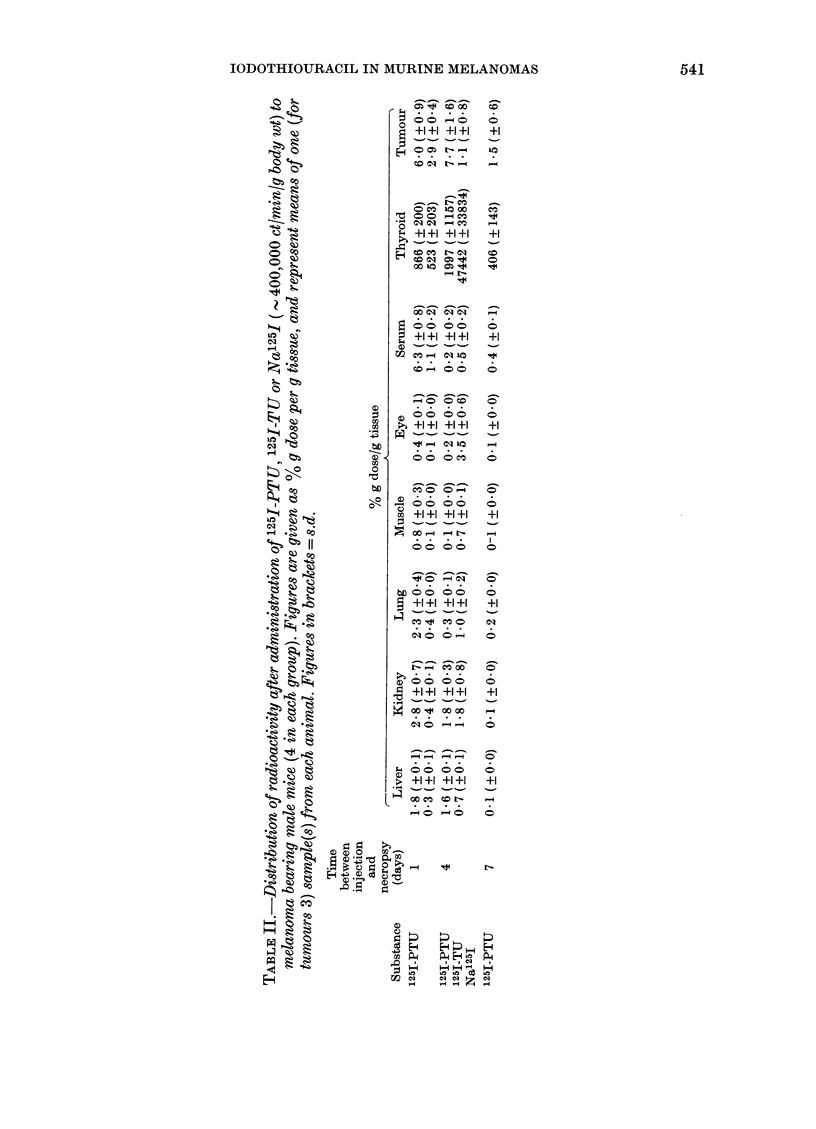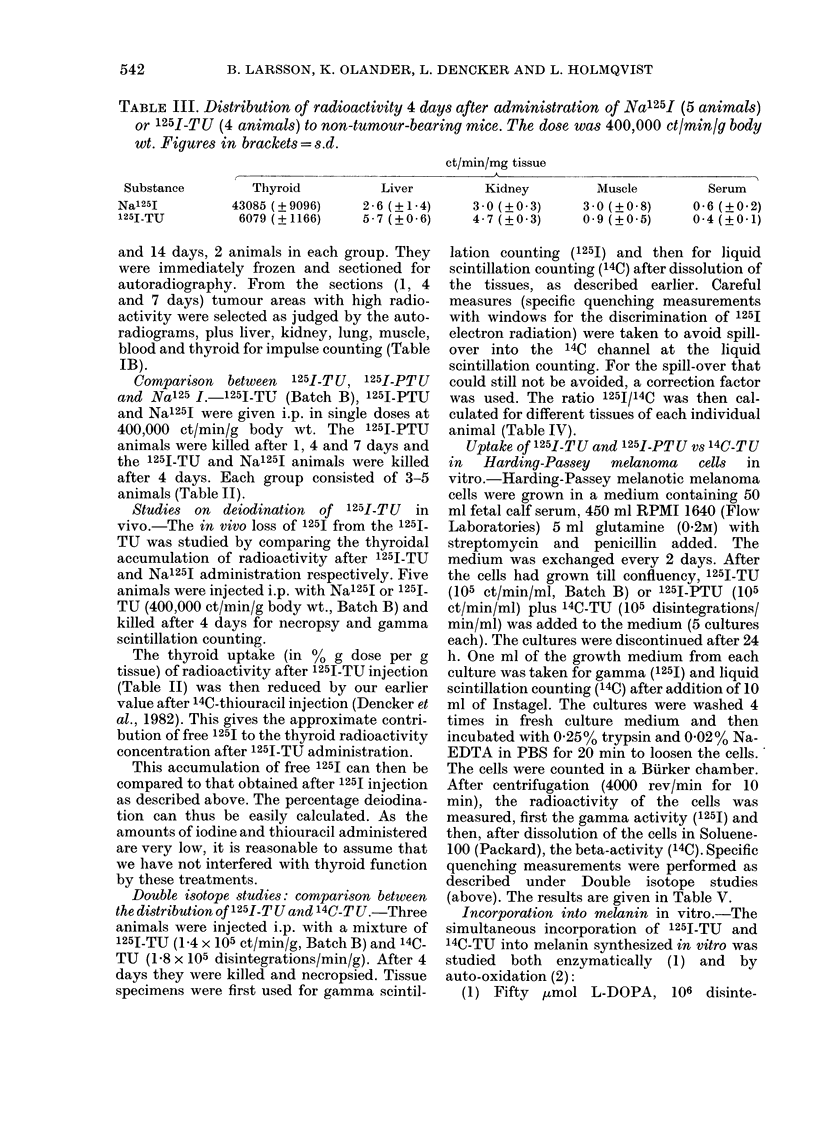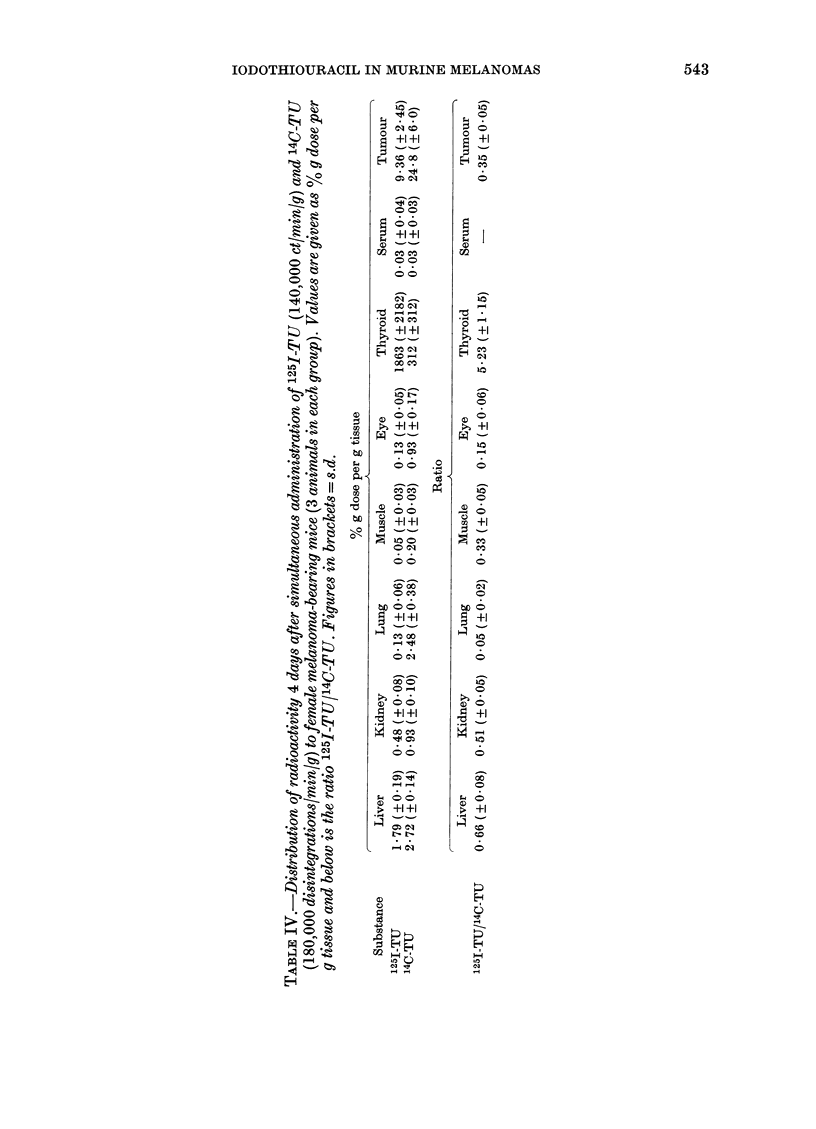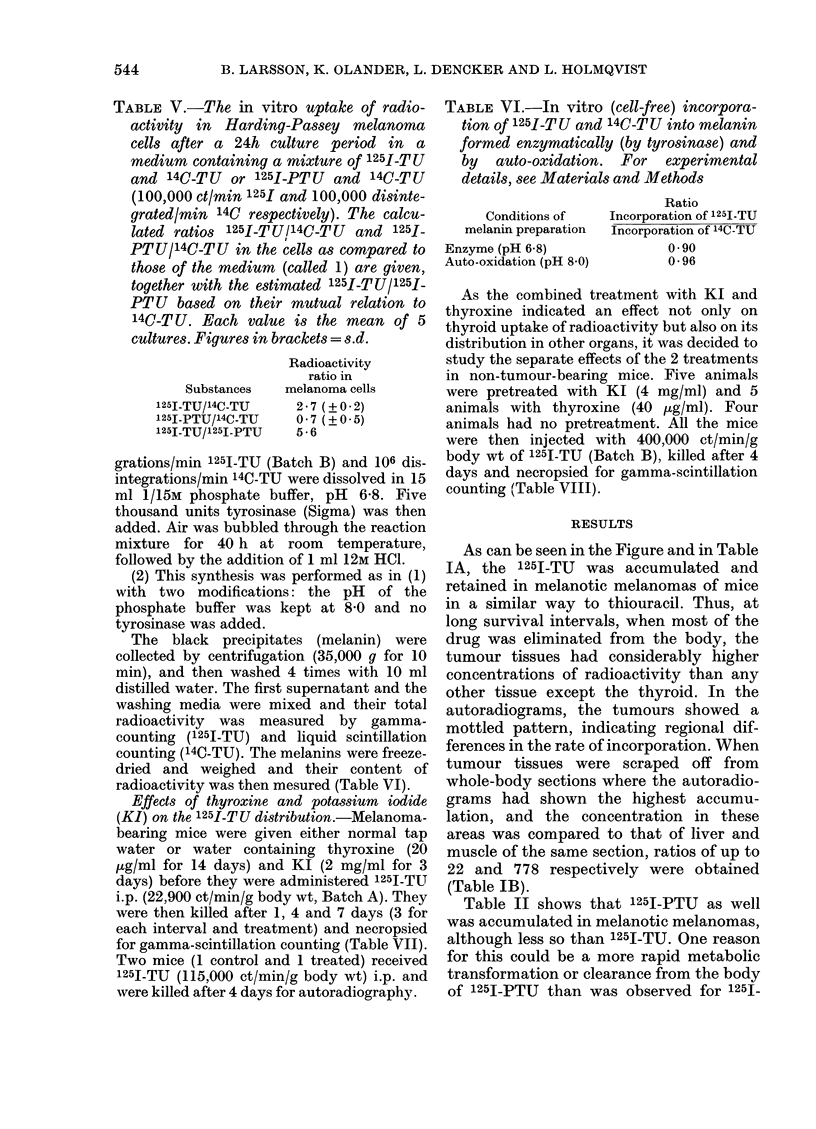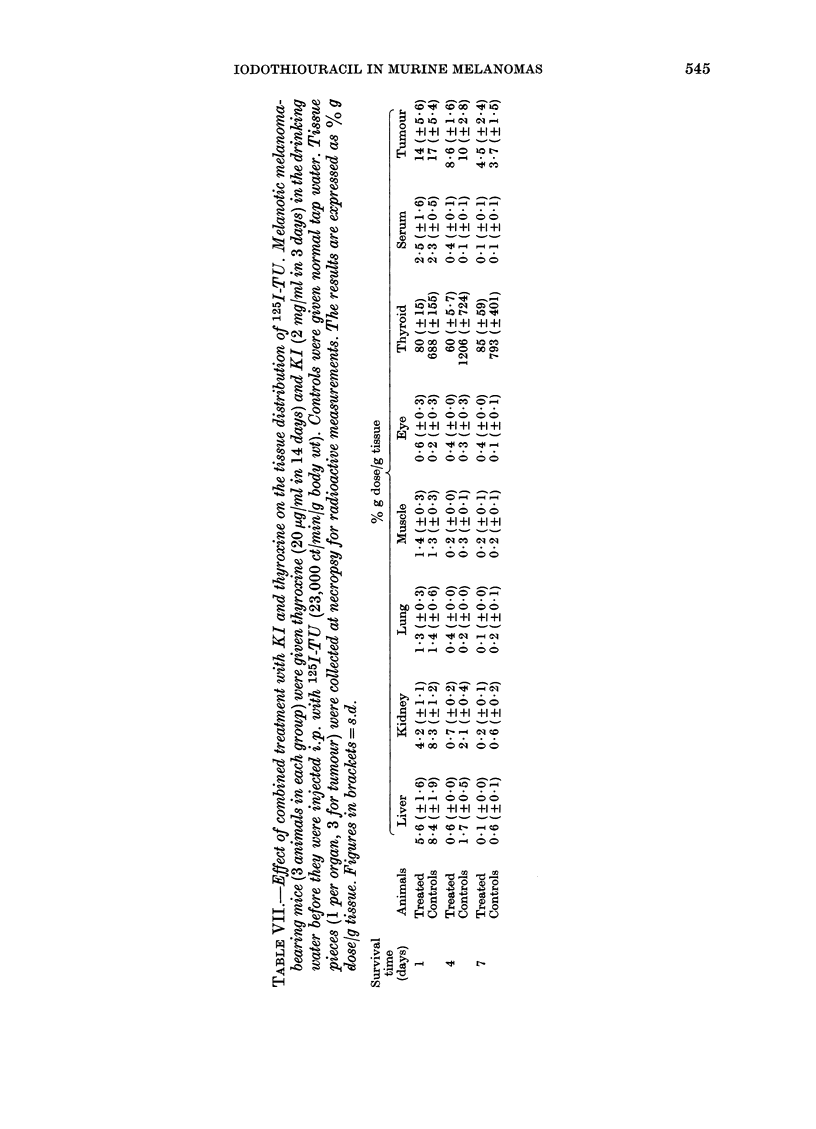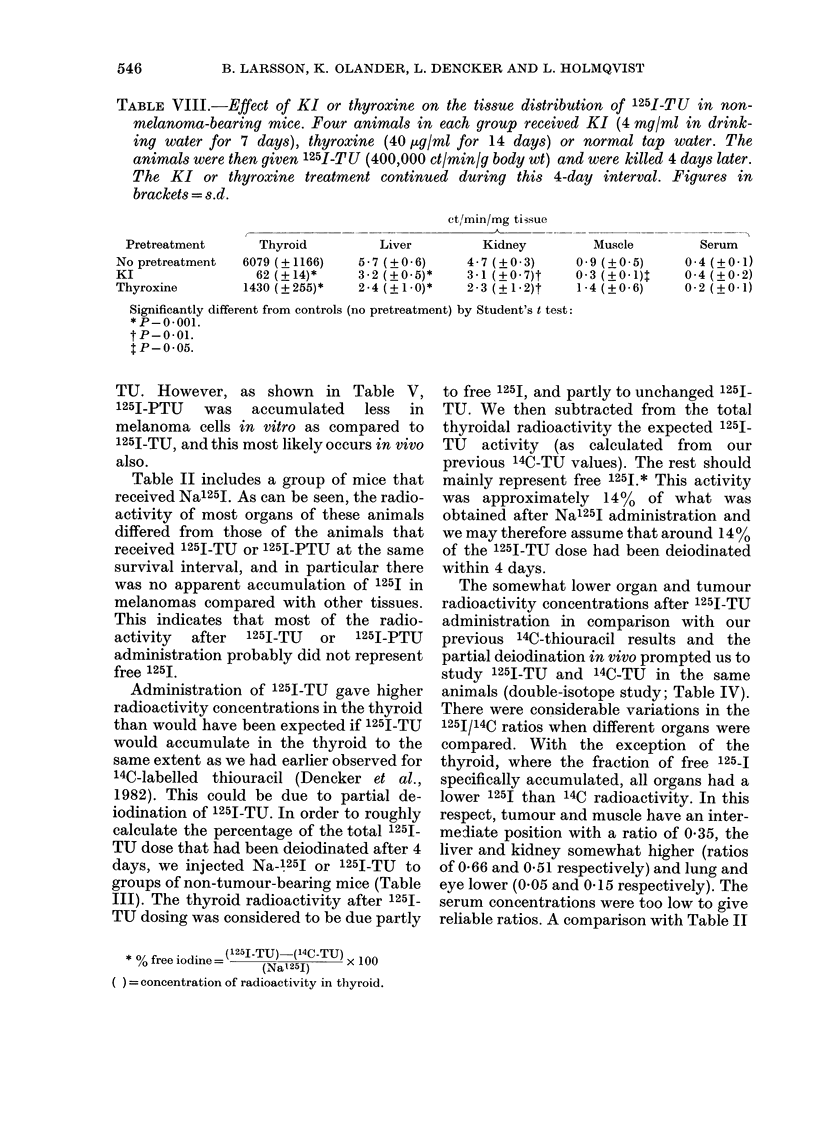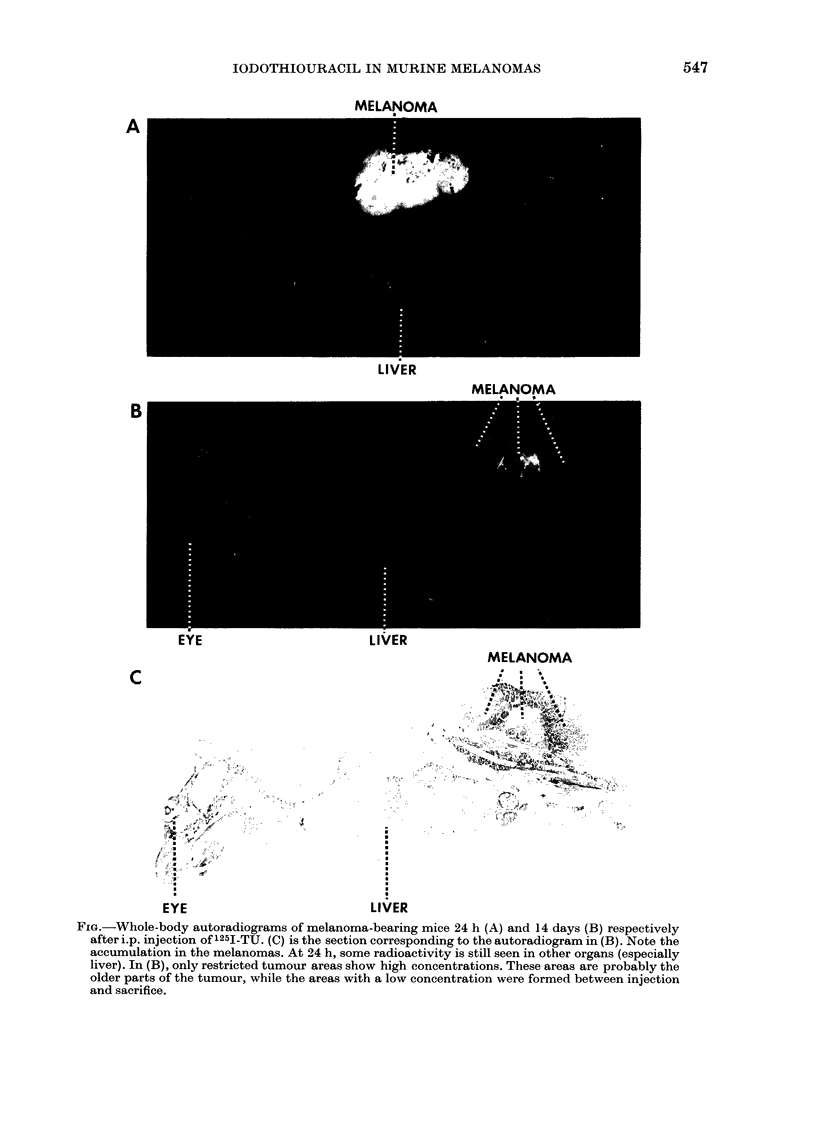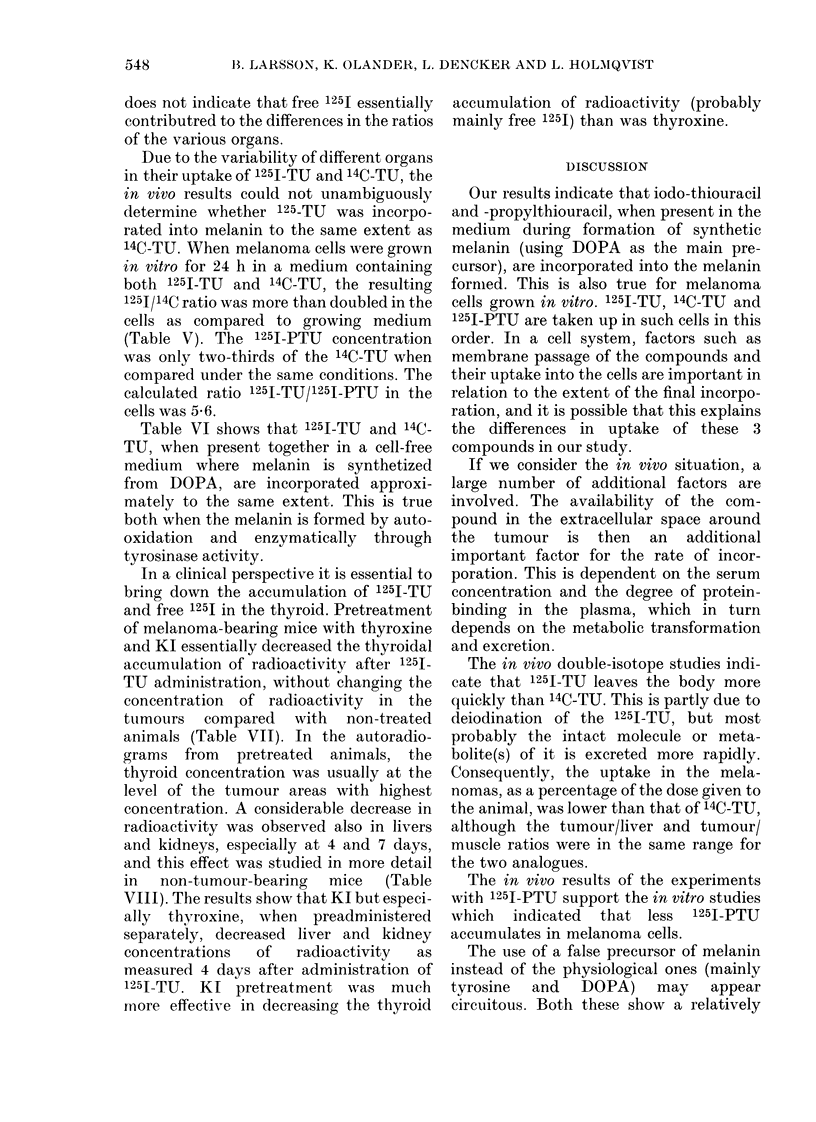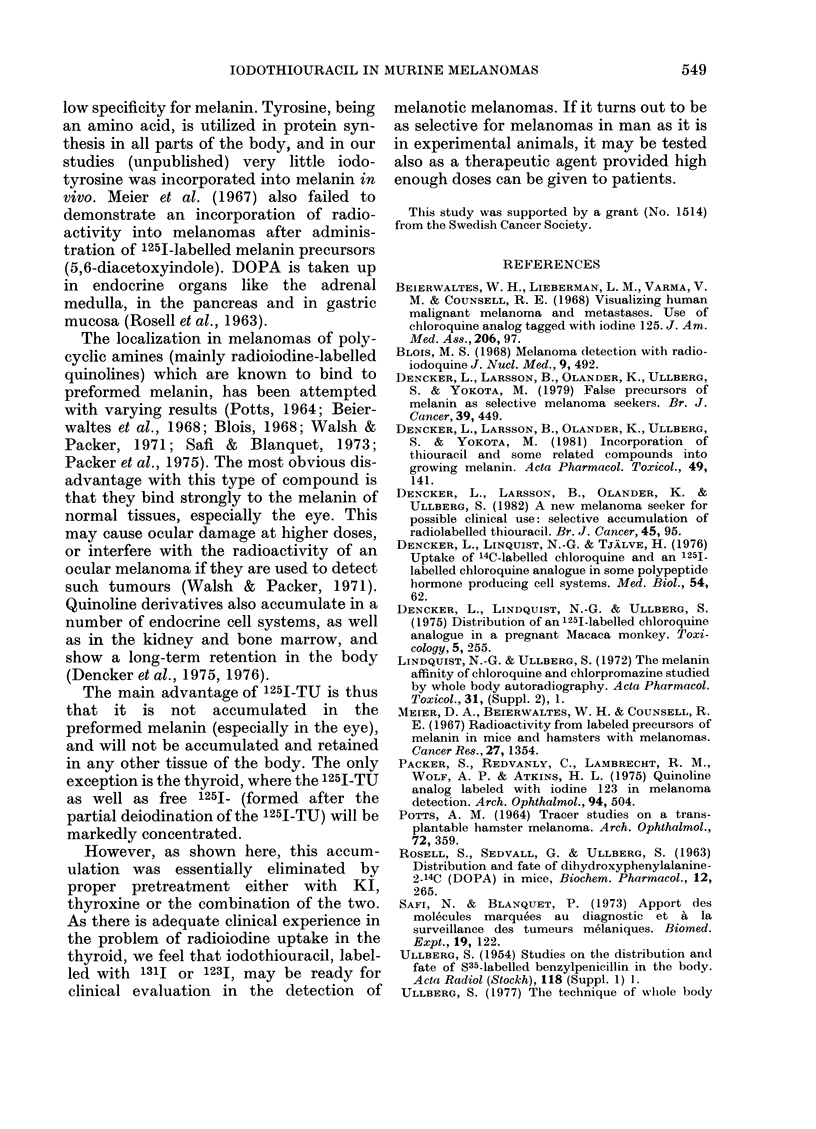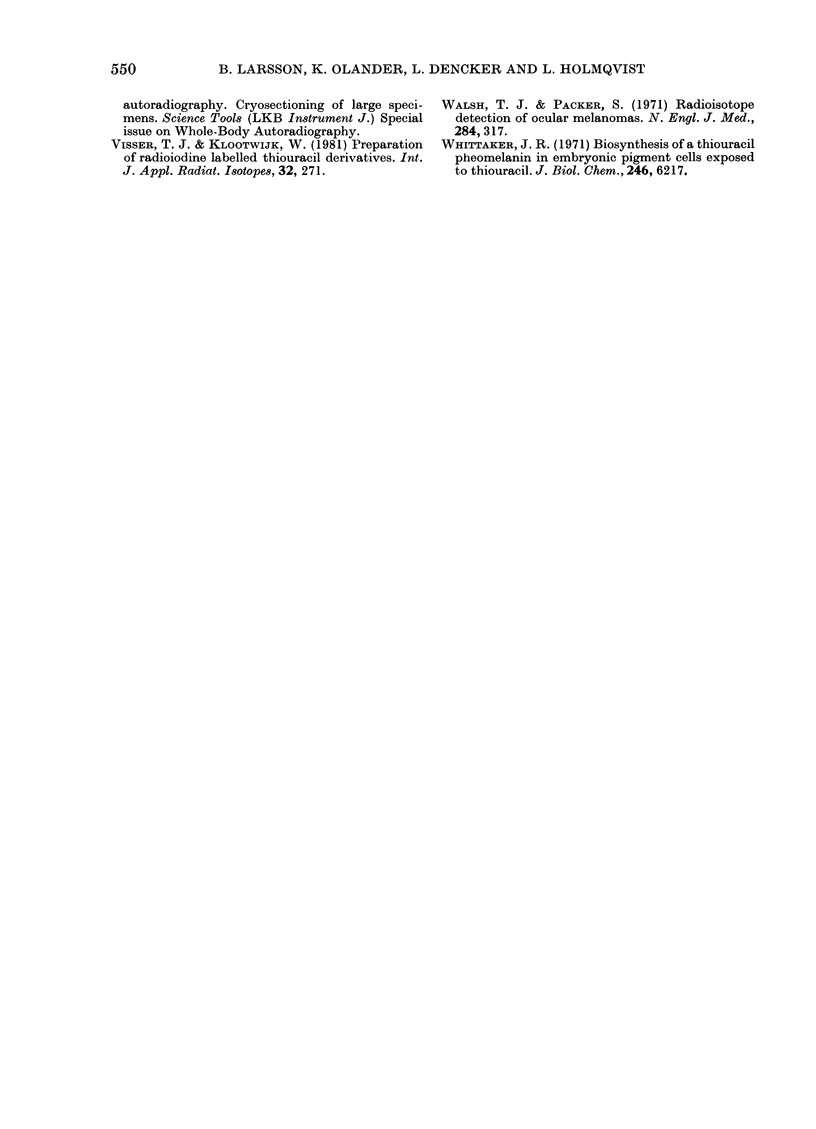# Accumulation of 125I-labelled thiouracil and propylthiouracil in murine melanotic melanomas.

**DOI:** 10.1038/bjc.1982.238

**Published:** 1982-10

**Authors:** B. Larsson, K. Olander, L. Dencker, L. Holmqvist

## Abstract

**Images:**


Br. J. Cancer (1982) 46, 538

### ACCUMULATION OF $^{125}I$-LABELLED THIOURACIL AND PROPYLTHIOURACIL IN MURINE MELANOTIC MELANOMAS

B. LARSSON, K. OLANDER, L. DENCKER AND L. HOLMQVIST*

*From the Department of Toxicology, University of Uppsala, Biomedical Centre, Box 573, S-751 23, and *AB Fortia, Virdinglaboratoriet, Box 604, S-751 25 Uppsala, Sweden*

Received 5 May 1982 Accepted 25 May 1982

**Summary.—We have shown that thioamides are incorporated as false precursors into melanin during its synthesis. To be clinically useful in the diagnosis or therapy of melanotic melanomas, they would have to be tagged with an appropriate isotope or possibly a cytotoxic moiety. $^{125}I$-Thiouracil ($^{125}I$-TU) is here shown to be accumulated in the melanin of melanotic melanomas transplanted into mice in a similar way as is $^{14}C$-thiouracil ($^{14}C$-TU). $^{125}I$-TU gives tumour/liver and tumour/muscle ratios up to 22 and 778 respectively, at 4 days after administration. $^{125}I$-TU is accumulated by melanoma cells *in vitro* more effectively than $^{14}C$-TU ($^{125}I$-TU/$^{14}C$-TU, 2·7), while the *in vivo* accumulation into melanomas is slightly lower for $^{125}I$-TU as compared to $^{14}C$-TU ($^{125}I$-TU/$^{14}C$-TU, 0·35). This appears to be due to a partial deiodination ($<14\%$ of the dose within 4 days) and probably a more rapid excretion of $^{125}I$-TU or its metabolite(s). The accumulation of radioactivity in the thyroid can essentially be eliminated by pretreatment with potassium iodide and/or thyroxine. $^{125}I$-Propylthiouracil is also accumulated in melanotic melanoma cells *in vivo* and *in vitro*, but at a lower level than in $^{125}I$-TU and $^{14}C$-TU.**

IN EARLIER STUDIES it was shown that thiouracil and related thioamides, in addition to their accumulation in the thyroid, are also incorporated into melanin when this is synthesized, *e.g.* in the eyes of foetuses or young animals and in melanin-producing tumours (Whittaker, 1971; Dencker *et al.*, 1979, 1981, 1982). Unlike drugs such as chloroquine and chlorpromazine (Lindquist & Ullberg, 1972), thiouracil does not bind to preformed melanin. If one is interested in finding drugs that might be clinically useful in localizing malignant melanomas, which usually have a high rate of melanin synthesis, then thioamides may be the drugs of choice.

To be clinically useful in this respect, the thioamides would have to be tagged with a gamma-emitting isotope with a relatively short half-life. Even if a number of different isotopes may prove useful, we have so far considered it most relevant to work with iodine, for which a number of isotopes are available.

The aim of this paper is to determine whether thiouracil labelled with iodine-125 is incorporated into melanotic melanoma cells *in vitro* and *in vivo* similarly to thiouracil. In addition we have studied the effects of potassium iodide and thyroxine on the uptake of radioactivity in the thyroid after $^{125}I$-thiouracil dosing. We have been able to determine approximately the extent of deiodination of $^{125}I$-thiouracil *in vivo*. Finally we have also studied the distribution of $^{125}I$-labelled propylthiouracil.

### MATERIALS AND METHODS

### *Chemicals*

2-Thiouracil (TU) was purchased from Aldrich-Europe, Belgium, and 6-n-propyl-2-thiouracil (PTU) from Sigma, St Louis, MO, U.S.A. $Na^{125}I$ (approx. 15·6 mCi/$\mu$g/$I^-$) was obtained from the Radiochemical Centre, Amersham, U.K. Other chemicals used in the study were of analytical grade and purchased from regular commercial sources.

*Radioiodination*

*5-$^{125}$I-2-Thiouracil* ($^{125}$*I-TU*).—Two batches were prepared independently by the chloramine-T method:

Batch A: To 1·7 mCi $^{125}I^-$(6 $\mu$l) was added 0·2 $\mu$g TU in 20 $\mu$l 0·2M phosphate buffer (pH 7.0) and 5 $\mu$l 6mM chloramine-T (in 0·2M phosphate buffer; pH 7·0). The mixture was thoroughly shaken. The reaction was allowed to proceed for 1 h after which 20 $\mu$l 0·1M sodium metabisulphite, 50 $\mu$l 0·1M carrier KI and 200 $\mu$l 0·05M phosphate buffer (pH 7·4) were added. The mixture was transferred to a small column of Sephadex G25 and eluted with 0·05M phosphate buffer (pH 7·4). Four different peaks were obtained (for further analysis see Results).

Batch B: This radioiodination was performed essentially as described by Visser & Klootwijk (1981). The main deviation was that the labelling was made at pH 7·5 (instead of 3·3), in order to increase the yield.

To 1 mCi $^{125}I^-$ (10 $\mu$l) was added 10 $\mu$l 0·5M sodium acetate (pH 8·8), 2 $\mu$g TU in 10 $\mu$l ethanol and 20 $\mu$g chloramine-T in 10 $\mu$l 0·01M phosphate buffer (pH 7·4). The final pH of the mixture was 7·5. After 10 min, the reaction was stopped by the addition of 100 $\mu$g sodium metabisulphite in 50 $\mu$l 0·1M acetic acid. The separation of $^{125}$I-TU from the mixture was made on Sephadex G10 in a small Pasteur pipette. Twenty-mM acetic acid containing 0·1mM dithiothreitol was used for elution and storage. Two peaks were obtained: unreacted $^{125}I^-$ and $^{125}$I-TU (60%). This is in agreement with the results of Visser & Klootwijk (1981). The specific activity of the $^{125}$I-TU was calculated to be higher than $10^3$ Ci/mmol.

*5-$^{125}$I-6-n-Propyl-2-thiouracil* ($^{125}$*I-PTU*).—PTU was radioiodinated in the same way as Batch B of TU.

*Animals*

Melanotic Harding-Passey tumours (obtained from AB Leo, Helsingborg, Sweden), were transplanted into DBA mice. Thus small pieces of the melanomas from non-necrotic areas were passed through a mesh into balanced salt solution (BSS). The cell suspension was then injected s.c. into the dorsum. When the tumours reached ½ in in diameter, the mice were used for the different experiments. They were then administered single i.p. doses of the drugs to be studied and were killed at different intervals after the injection (usually at 1, 4 and 7 days), by inhalation of carbon dioxide.

*Whole-body autoradiography*

After killing, the animals were mounted in a gel of carboxylmethyl cellulose and frozen in hexane, cooled with dry ice. Sections attached on to tape (No. 810, Minnesota Mining and Manufacturing Co., U.S.A.) were taken at different levels of the body (Ullberg, 1954, 1977). The sections were then freeze-dried and opposed against X-ray film for autoradiography (section thickness; 20 and 60 $\mu$m).

*Scintillation-counting experiments*

Quantitative measurements were performed in different ways. Either tissues were scraped off from thick (200$\mu$m) tape-fastened sections or were dissected out at necropsy directly after killing. For $^{125}$I, the tissues were measured in a Packard Auto-Gamma scintillation spectrometer. For $^{14}$C, melanin-containing tissues were dissolved in Soluene-100 (Packard), bleached by the addition of 0·2 ml isopropanol and 0·2 ml of 35% $H_2O_2$ and incubated at 40°C for 30 min, after which 15 ml of Instagel was added. To the serum samples was added 1 ml of water and 10 ml of Instagel. Remaining tissues were dissolved in Soluene-350 (Packard), after which 10 ml of scintillation fluid (4·9 g PPO, 0·1 g dimethyl POPOP per 1 toluene) was added. The specimens were counted in a Packard Tricarb 460CD liquid scintillation spectrometer with the use of an external standard.

*Experimental groups*

*Distribution studies of* $^{125}$*I-TU*.—Tumour-bearing mice, 3–12 animals in each group, received single i.p. injections of varying radio-doses of $^{125}$I-TU (Batch A) and were killed after 1, 4 and 7 days. Pieces of tissues from a number of organs and 2–3 tumour pieces were excised from each animal. The radioactivity was measured and calculated as the percentage of the given dose recovered per g of tissue (% g dose per g tissue; Table IA). Eight melanoma-bearing mice received single i.p. injections of $^{125}$I-TU (1,170,000 ct/min/g body wt. Batch B) and were killed after 1, 4, 7

TABLE III. *Distribution of radioactivity 4 days after administration of $Na^{125}I$ (5 animals) or $^{125}I$-TU (4 animals) to non-tumour-bearing mice. The dose was 400,000 ct/min/g body wt. Figures in brackets = s.d.*

| | ct/min/mg tissue | | | | |
|---|---|---|---|---|---|
| Substance | Thyroid | Liver | Kidney | Muscle | Serum |
| $Na^{125}I$ | 43085 (±9096) | 2·6 (±1·4) | 3·0 (±0·3) | 3·0 (±0·8) | 0·6 (±0·2) |
| $^{125}I$-TU | 6079 (±1166) | 5·7 (±0·6) | 4·7 (±0·3) | 0·9 (±0·5) | 0·4 (±0·1) |

and 14 days, 2 animals in each group. They were immediately frozen and sectioned for autoradiography. From the sections (1, 4 and 7 days) tumour areas with high radioactivity were selected as judged by the autoradiograms, plus liver, kidney, lung, muscle, blood and thyroid for impulse counting (Table IB).

*Comparison between $^{125}I$-TU, $^{125}I$-PTU and $Na^{125}I$.*—$^{125}I$-TU (Batch B), $^{125}I$-PTU and $Na^{125}I$ were given i.p. in single doses at 400,000 ct/min/g body wt. The $^{125}I$-PTU animals were killed after 1, 4 and 7 days and the $^{125}I$-TU and $Na^{125}I$ animals were killed after 4 days. Each group consisted of 3–5 animals (Table II).

*Studies on deiodination of $^{125}I$-TU* in vivo.—The *in vivo* loss of $^{125}I$ from the $^{125}I$-TU was studied by comparing the thyroidal accumulation of radioactivity after $^{125}I$-TU and $Na^{125}I$ administration respectively. Five animals were injected i.p. with $Na^{125}I$ or $^{125}I$-TU (400,000 ct/min/g body wt., Batch B) and killed after 4 days for necropsy and gamma scintillation counting.

The thyroid uptake (in % g dose per g tissue) of radioactivity after $^{125}I$-TU injection (Table II) was then reduced by our earlier value after $^{14}C$-thiouracil injection (Dencker *et al.*, 1982). This gives the approximate contribution of free $^{125}I$ to the thyroid radioactivity concentration after $^{125}I$-TU administration.

This accumulation of free $^{125}I$ can then be compared to that obtained after $^{125}I$ injection as described above. The percentage deiodination can thus be easily calculated. As the amounts of iodine and thiouracil administered are very low, it is reasonable to assume that we have not interfered with thyroid function by these treatments.

*Double isotope studies: comparison between the distribution of $^{125}I$-TU and $^{14}C$-TU.*—Three animals were injected i.p. with a mixture of $^{125}I$-TU ($1·4 \times 10^5$ ct/min/g, Batch B) and $^{14}C$-TU ($1·8 \times 10^5$ disintegrations/min/g). After 4 days they were killed and necropsied. Tissue specimens were first used for gamma scintillation counting ($^{125}I$) and then for liquid scintillation counting ($^{14}C$) after dissolution of the tissues, as described earlier. Careful measures (specific quenching measurements with windows for the discrimination of $^{125}I$ electron radiation) were taken to avoid spill-over into the $^{14}C$ channel at the liquid scintillation counting. For the spill-over that could still not be avoided, a correction factor was used. The ratio $^{125}I/^{14}C$ was then calculated for different tissues of each individual animal (Table IV).

*Uptake of $^{125}I$-TU and $^{125}I$-PTU vs $^{14}C$-TU in Harding-Passey melanoma cells* in vitro.—Harding-Passey melanotic melanoma cells were grown in a medium containing 50 ml fetal calf serum, 450 ml RPMI 1640 (Flow Laboratories) 5 ml glutamine (0·2M) with streptomycin and penicillin added. The medium was exchanged every 2 days. After the cells had grown till confluency, $^{125}I$-TU ($10^5$ ct/min/ml, Batch B) or $^{125}I$-PTU ($10^5$ ct/min/ml) plus $^{14}C$-TU ($10^5$ disintegrations/min/ml) was added to the medium (5 cultures each). The cultures were discontinued after 24 h. One ml of the growth medium from each culture was taken for gamma ($^{125}I$) and liquid scintillation counting ($^{14}C$) after addition of 10 ml of Instagel. The cultures were washed 4 times in fresh culture medium and then incubated with 0·25% trypsin and 0·02% Na-EDTA in PBS for 20 min to loosen the cells. The cells were counted in a Bürker chamber. After centrifugation (4000 rev/min for 10 min), the radioactivity of the cells was measured, first the gamma activity ($^{125}I$) and then, after dissolution of the cells in Soluene-100 (Packard), the beta-activity ($^{14}C$). Specific quenching measurements were performed as described under Double isotope studies (above). The results are given in Table V.

*Incorporation into melanin* in vitro.—The simultaneous incorporation of $^{125}I$-TU and $^{14}C$-TU into melanin synthesized *in vitro* was studied both enzymatically (1) and by auto-oxidation (2):

(1) Fifty $\mu$mol L-DOPA, $10^6$ disinte-

TABLE V.—*The* in vitro *uptake of radioactivity in Harding-Passey melanoma cells after a 24h culture period in a medium containing a mixture of* $^{125}$*I-TU and* $^{14}$*C-TU or* $^{125}$*I-PTU and* $^{14}$*C-TU* (100,000 *ct/min* $^{125}$*I and* 100,000 *disintegrated/min* $^{14}$*C respectively*). *The calculated ratios* $^{125}$*I-TU/*$^{14}$*C-TU and* $^{125}$*I-PTU/*$^{14}$*C-TU in the cells as compared to those of the medium* (*called* 1) *are given, together with the estimated* $^{125}$*I-TU/*$^{125}$*I-PTU based on their mutual relation to* $^{14}$*C-TU. Each value is the mean of* 5 *cultures. Figures in brackets = s.d.*

| Substances | Radioactivity ratio in melanoma cells |
|---|---|
| $^{125}$I-TU/$^{14}$C-TU | 2·7 (±0·2) |
| $^{125}$I-PTU/$^{14}$C-TU | 0·7 (±0·5) |
| $^{125}$I-TU/$^{125}$I-PTU | 5·6 |

grations/min $^{125}$I-TU (Batch B) and $10^6$ disintegrations/min $^{14}$C-TU were dissolved in 15 ml 1/15M phosphate buffer, pH 6·8. Five thousand units tyrosinase (Sigma) was then added. Air was bubbled through the reaction mixture for 40 h at room temperature, followed by the addition of 1 ml 12M HCl.

(2) This synthesis was performed as in (1) with two modifications: the pH of the phosphate buffer was kept at 8·0 and no tyrosinase was added.

The black precipitates (melanin) were collected by centrifugation (35,000 *g* for 10 min), and then washed 4 times with 10 ml distilled water. The first supernatant and the washing media were mixed and their total radioactivity was measured by gamma-counting ($^{125}$I-TU) and liquid scintillation counting ($^{14}$C-TU). The melanins were freeze-dried and weighed and their content of radioactivity was then mesured (Table VI).

*Effects of thyroxine and potassium iodide* (*KI*) *on the* $^{125}$*I-TU distribution.*—Melanoma-bearing mice were given either normal tap water or water containing thyroxine (20 μg/ml for 14 days) and KI (2 mg/ml for 3 days) before they were administered $^{125}$I-TU i.p. (22,900 ct/min/g body wt, Batch A). They were then killed after 1, 4 and 7 days (3 for each interval and treatment) and necropsied for gamma-scintillation counting (Table VII). Two mice (1 control and 1 treated) received $^{125}$I-TU (115,000 ct/min/g body wt) i.p. and were killed after 4 days for autoradiography.

TABLE VI.—In vitro (*cell-free*) *incorporation of* $^{125}$*I-TU and* $^{14}$*C-TU into melanin formed enzymatically* (*by tyrosinase*) *and by auto-oxidation. For experimental details, see Materials and Methods*

| Conditions of melanin preparation | Ratio: Incorporation of $^{125}$I-TU / Incorporation of $^{14}$C-TU |
|---|---|
| Enzyme (pH 6·8) | 0·90 |
| Auto-oxidation (pH 8·0) | 0·96 |

As the combined treatment with KI and thyroxine indicated an effect not only on thyroid uptake of radioactivity but also on its distribution in other organs, it was decided to study the separate effects of the 2 treatments in non-tumour-bearing mice. Five animals were pretreated with KI (4 mg/ml) and 5 animals with thyroxine (40 μg/ml). Four animals had no pretreatment. All the mice were then injected with 400,000 ct/min/g body wt of $^{125}$I-TU (Batch B), killed after 4 days and necropsied for gamma-scintillation counting (Table VIII).

### RESULTS

As can be seen in the Figure and in Table IA, the $^{125}$I-TU was accumulated and retained in melanotic melanomas of mice in a similar way to thiouracil. Thus, at long survival intervals, when most of the drug was eliminated from the body, the tumour tissues had considerably higher concentrations of radioactivity than any other tissue except the thyroid. In the autoradiograms, the tumours showed a mottled pattern, indicating regional differences in the rate of incorporation. When tumour tissues were scraped off from whole-body sections where the autoradiograms had shown the highest accumulation, and the concentration in these areas was compared to that of liver and muscle of the same section, ratios of up to 22 and 778 respectively were obtained (Table IB).

Table II shows that $^{125}$I-PTU as well was accumulated in melanotic melanomas, although less so than $^{125}$I-TU. One reason for this could be a more rapid metabolic transformation or clearance from the body of $^{125}$I-PTU than was observed for $^{125}$I-

TABLE VIII.—*Effect of KI or thyroxine on the tissue distribution of $^{125}$I-TU in non-melanoma-bearing mice. Four animals in each group received KI (4 mg/ml in drinking water for 7 days), thyroxine (40 µg/ml for 14 days) or normal tap water. The animals were then given $^{125}$I-TU (400,000 ct/min/g body wt) and were killed 4 days later. The KI or thyroxine treatment continued during this 4-day interval. Figures in brackets = s.d.*

| | ct/min/mg tissue | | | | |
|---|---|---|---|---|---|
| Pretreatment | Thyroid | Liver | Kidney | Muscle | Serum |
| No pretreatment | 6079 (±1166) | 5·7 (±0·6) | 4·7 (±0·3) | 0·9 (±0·5) | 0·4 (±0·1) |
| KI | 62 (±14)* | 3·2 (±0·5)* | 3·1 (±0·7)† | 0·3 (±0·1)‡ | 0·4 (±0·2) |
| Thyroxine | 1430 (±255)* | 2·4 (±1·0)* | 2·3 (±1·2)† | 1·4 (±0·6) | 0·2 (±0·1) |

Significantly different from controls (no pretreatment) by Student's $t$ test:
* $P-0·001$.
† $P-0·01$.
‡ $P-0·05$.

TU. However, as shown in Table V, $^{125}$I-PTU was accumulated less in melanoma cells *in vitro* as compared to $^{125}$I-TU, and this most likely occurs *in vivo* also.

Table II includes a group of mice that received Na$^{125}$I. As can be seen, the radioactivity of most organs of these animals differed from those of the animals that received $^{125}$I-TU or $^{125}$I-PTU at the same survival interval, and in particular there was no apparent accumulation of $^{125}$I in melanomas compared with other tissues. This indicates that most of the radioactivity after $^{125}$I-TU or $^{125}$I-PTU administration probably did not represent free $^{125}$I.

Administration of $^{125}$I-TU gave higher radioactivity concentrations in the thyroid than would have been expected if $^{125}$I-TU would accumulate in the thyroid to the same extent as we had earlier observed for $^{14}$C-labelled thiouracil (Dencker *et al.*, 1982). This could be due to partial deiodination of $^{125}$I-TU. In order to roughly calculate the percentage of the total $^{125}$I-TU dose that had been deiodinated after 4 days, we injected Na-$^{125}$I or $^{125}$I-TU to groups of non-tumour-bearing mice (Table III). The thyroid radioactivity after $^{125}$I-TU dosing was considered to be due partly to free $^{125}$I, and partly to unchanged $^{125}$I-TU. We then subtracted from the total thyroidal radioactivity the expected $^{125}$I-TU activity (as calculated from our previous $^{14}$C-TU values). The rest should mainly represent free $^{125}$I.* This activity was approximately 14% of what was obtained after Na$^{125}$I administration and we may therefore assume that around 14% of the $^{125}$I-TU dose had been deiodinated within 4 days.

The somewhat lower organ and tumour radioactivity concentrations after $^{125}$I-TU administration in comparison with our previous $^{14}$C-thiouracil results and the partial deiodination *in vivo* prompted us to study $^{125}$I-TU and $^{14}$C-TU in the same animals (double-isotope study; Table IV). There were considerable variations in the $^{125}$I/$^{14}$C ratios when different organs were compared. With the exception of the thyroid, where the fraction of free $^{125}$-I specifically accumulated, all organs had a lower $^{125}$I than $^{14}$C radioactivity. In this respect, tumour and muscle have an intermediate position with a ratio of 0·35, the liver and kidney somewhat higher (ratios of 0·66 and 0·51 respectively) and lung and eye lower (0·05 and 0·15 respectively). The serum concentrations were too low to give reliable ratios. A comparison with Table II

$$* \ \% \text{ free iodine} = \frac{(^{125}\text{I-TU})—(^{14}\text{C-TU})}{(\text{Na}^{125}\text{I})} \times 100$$

( ) = concentration of radioactivity in thyroid.

FIG.—Whole-body autoradiograms of melanoma-bearing mice 24 h (A) and 14 days (B) respectively after i.p. injection of $^{125}$I-TU. (C) is the section corresponding to the autoradiogram in (B). Note the accumulation in the melanomas. At 24 h, some radioactivity is still seen in other organs (especially liver). In (B), only restricted tumour areas show high concentrations. These areas are probably the older parts of the tumour, while the areas with a low concentration were formed between injection and sacrifice.

does not indicate that free $^{125}I$ essentially contributred to the differences in the ratios of the various organs.

Due to the variability of different organs in their uptake of $^{125}I$-TU and $^{14}C$-TU, the *in vivo* results could not unambiguously determine whether $^{125}$-TU was incorporated into melanin to the same extent as $^{14}C$-TU. When melanoma cells were grown *in vitro* for 24 h in a medium containing both $^{125}I$-TU and $^{14}C$-TU, the resulting $^{125}I/^{14}C$ ratio was more than doubled in the cells as compared to growing medium (Table V). The $^{125}I$-PTU concentration was only two-thirds of the $^{14}C$-TU when compared under the same conditions. The calculated ratio $^{125}I$-TU/$^{125}I$-PTU in the cells was 5·6.

Table VI shows that $^{125}I$-TU and $^{14}C$-TU, when present together in a cell-free medium where melanin is synthetized from DOPA, are incorporated approximately to the same extent. This is true both when the melanin is formed by autooxidation and enzymatically through tyrosinase activity.

In a clinical perspective it is essential to bring down the accumulation of $^{125}I$-TU and free $^{125}I$ in the thyroid. Pretreatment of melanoma-bearing mice with thyroxine and KI essentially decreased the thyroidal accumulation of radioactivity after $^{125}I$-TU administration, without changing the concentration of radioactivity in the tumours compared with non-treated animals (Table VII). In the autoradiograms from pretreated animals, the thyroid concentration was usually at the level of the tumour areas with highest concentration. A considerable decrease in radioactivity was observed also in livers and kidneys, especially at 4 and 7 days, and this effect was studied in more detail in non-tumour-bearing mice (Table VIII). The results show that KI but especially thyroxine, when preadministered separately, decreased liver and kidney concentrations of radioactivity as measured 4 days after administration of $^{125}I$-TU. KI pretreatment was much more effective in decreasing the thyroid accumulation of radioactivity (probably mainly free $^{125}I$) than was thyroxine.

### DISCUSSION

Our results indicate that iodo-thiouracil and -propylthiouracil, when present in the medium during formation of synthetic melanin (using DOPA as the main precursor), are incorporated into the melanin formed. This is also true for melanoma cells grown *in vitro*. $^{125}I$-TU, $^{14}C$-TU and $^{125}I$-PTU are taken up in such cells in this order. In a cell system, factors such as membrane passage of the compounds and their uptake into the cells are important in relation to the extent of the final incorporation, and it is possible that this explains the differences in uptake of these 3 compounds in our study.

If we consider the *in vivo* situation, a large number of additional factors are involved. The availability of the compound in the extracellular space around the tumour is then an additional important factor for the rate of incorporation. This is dependent on the serum concentration and the degree of protein-binding in the plasma, which in turn depends on the metabolic transformation and excretion.

The *in vivo* double-isotope studies indicate that $^{125}I$-TU leaves the body more quickly than $^{14}C$-TU. This is partly due to deiodination of the $^{125}I$-TU, but most probably the intact molecule or metabolite(s) of it is excreted more rapidly. Consequently, the uptake in the melanomas, as a percentage of the dose given to the animal, was lower than that of $^{14}C$-TU, although the tumour/liver and tumour/muscle ratios were in the same range for the two analogues.

The *in vivo* results of the experiments with $^{125}I$-PTU support the *in vitro* studies which indicated that less $^{125}I$-PTU accumulates in melanoma cells.

The use of a false precursor of melanin instead of the physiological ones (mainly tyrosine and DOPA) may appear circuitous. Both these show a relatively low specificity for melanin. Tyrosine, being an amino acid, is utilized in protein synthesis in all parts of the body, and in our studies (unpublished) very little iodotyrosine was incorporated into melanin *in vivo*. Meier *et al.* (1967) also failed to demonstrate an incorporation of radioactivity into melanomas after administration of $^{125}$I-labelled melanin precursors (5,6-diacetoxyindole). DOPA is taken up in endocrine organs like the adrenal medulla, in the pancreas and in gastric mucosa (Rosell *et al.*, 1963).

The localization in melanomas of polycyclic amines (mainly radioiodine-labelled quinolines) which are known to bind to preformed melanin, has been attempted with varying results (Potts, 1964; Beierwaltes *et al.*, 1968; Blois, 1968; Walsh & Packer, 1971; Safi & Blanquet, 1973; Packer *et al.*, 1975). The most obvious disadvantage with this type of compound is that they bind strongly to the melanin of normal tissues, especially the eye. This may cause ocular damage at higher doses, or interfere with the radioactivity of an ocular melanoma if they are used to detect such tumours (Walsh & Packer, 1971). Quinoline derivatives also accumulate in a number of endocrine cell systems, as well as in the kidney and bone marrow, and show a long-term retention in the body (Dencker *et al.*, 1975, 1976).

The main advantage of $^{125}$I-TU is thus that it is not accumulated in the preformed melanin (especially in the eye), and will not be accumulated and retained in any other tissue of the body. The only exception is the thyroid, where the $^{125}$I-TU as well as free $^{125}$I- (formed after the partial deiodination of the $^{125}$I-TU) will be markedly concentrated.

However, as shown here, this accumulation was essentially eliminated by proper pretreatment either with KI, thyroxine or the combination of the two. As there is adequate clinical experience in the problem of radioiodine uptake in the thyroid, we feel that iodothiouracil, labelled with $^{131}$I or $^{123}$I, may be ready for clinical evaluation in the detection of melanotic melanomas. If it turns out to be as selective for melanomas in man as it is in experimental animals, it may be tested also as a therapeutic agent provided high enough doses can be given to patients.

This study was supported by a grant (No. 1514) from the Swedish Cancer Society.

### REFERENCES

Beierwaltes, W. H., Lieberman, L. M., Varma, V. M. & Counsell, R. E. (1968) Visualizing human malignant melanoma and metastases. Use of chloroquine analog tagged with iodine 125. *J. Am. Med. Ass.*, **206**, 97.

Blois, M. S. (1968) Melanoma detection with radioiodoquine *J. Nucl. Med.*, **9**, 492.

Dencker, L., Larsson, B., Olander, K., Ullberg, S. & Yokota, M. (1979) False precursors of melanin as selective melanoma seekers. *Br. J. Cancer*, **39**, 449.

Dencker, L., Larsson, B., Olander, K., Ullberg, S. & Yokota, M. (1981) Incorporation of thiouracil and some related compounds into growing melanin. *Acta Pharmacol. Toxicol.*, **49**, 141.

Dencker, L., Larsson, B., Olander, K. & Ullberg, S. (1982) A new melanoma seeker for possible clinical use: selective accumulation of radiolabelled thiouracil. *Br. J. Cancer*, **45**, 95.

Dencker, L., Linquist, N.-G. & Tjälve, H. (1976) Uptake of $^{14}$C-labelled chloroquine and an $^{125}$I-labelled chloroquine analogue in some polypeptide hormone producing cell systems. *Med. Biol.*, **54**, 62.

Dencker, L., Lindquist, N.-G. & Ullberg, S. (1975) Distribution of an $^{125}$I-labelled chloroquine analogue in a pregnant Macaca monkey. *Toxicology*, **5**, 255.

Lindquist, N.-G. & Ullberg, S. (1972) The melanin affinity of chloroquine and chlorpromazine studied by whole body autoradiography. *Acta Pharmacol. Toxicol.*, **31**, (Suppl. 2), 1.

Meier, D. A., Beierwaltes, W. H. & Counsell, R. E. (1967) Radioactivity from labeled precursors of melanin in mice and hamsters with melanomas. *Cancer Res.*, **27**, 1354.

Packer, S., Redvanly, C., Lambrecht, R. M., Wolf, A. P. & Atkins, H. L. (1975) Quinoline analog labeled with iodine 123 in melanoma detection. *Arch. Ophthalmol.*, **94**, 504.

Potts, A. M. (1964) Tracer studies on a transplantable hamster melanoma. *Arch. Ophthalmol.*, **72**, 359.

Rosell, S., Sedvall, G. & Ullberg, S. (1963) Distribution and fate of dihydroxyphenylalanine-2-$^{14}$C (DOPA) in mice, *Biochem. Pharmacol.*, **12**, 265.

Safi, N. & Blanquet, P. (1973) Apport des molécules marquées au diagnostic et à la surveillance des tumeurs mélaniques. *Biomed. Expt.*, **19**, 122.

Ullberg, S. (1954) Studies on the distribution and fate of $S^{35}$-labelled benzylpenicillin in the body. *Acta Radiol* (*Stockh*), **118** (Suppl. 1) 1.

Ullberg, S. (1977) The technique of whole body autoradiography. Cryosectioning of large specimens. *Science Tools* (LKB *Instrument J.*) Special issue on Whole-Body Autoradiography.

VISSER, T. J. & KLOOTWIJK, W. (1981) Preparation of radioiodine labelled thiouracil derivatives. *Int. J. Appl. Radiat. Isotopes*, **32**, 271.

WALSH, T. J. & PACKER, S. (1971) Radioisotope detection of ocular melanomas. *N. Engl. J. Med.*, **284**, 317.

WHITTAKER, J. R. (1971) Biosynthesis of a thiouracil pheomelanin in embryonic pigment cells exposed to thiouracil. *J. Biol. Chem.*, **246**, 6217.